# The diagnostic accuracy of high *b*-value diffusion- and T_2_-weighted imaging for the detection of prostate cancer: a meta-analysis

**DOI:** 10.1007/s00261-017-1400-4

**Published:** 2017-11-24

**Authors:** Tom J. Syer, Keith C. Godley, Donnie Cameron, Paul N. Malcolm

**Affiliations:** 1 Norwich Medical School, Faculty of Medicine and Health Sciences, University of East Anglia, Norwich, Norfolk NR4 7TJ UK; 2 Radiology Department, Norfolk & Norwich University NHS Foundation Trust, Colney Lane, Norfolk Norwich, NR4 7UY UK

**Keywords:** Prostate cancer, Diffusion-weighted imaging, T_2_-weighted imaging, *b*-value, Meta-analysis

## Abstract

**Purpose:**

This study aims to investigate the role of diffusion-weighted imaging (DWI) and T_2_-weighted imaging (T_2_WI) in combination for the detection of prostate cancer, specifically assessing the role of high *b*-values (> 1000 s/mm^2^), with a systematic review and meta-analysis of the existing published data.

**Methods:**

The electronic databases MEDLINE, EMBASE, and OpenSIGLE were searched between inception and September 1, 2017. Eligible studies were those that reported the sensitivity and specificity of DWI and T_2_WI for the diagnosis of prostate cancer by visual assessment using a histopathologic reference standard. The QUADAS-2 critical appraisal tool was used to assess the quality of included studies. A meta-analysis with pooling of sensitivity, specificity, likelihood, and diagnostic odds ratios was undertaken, and a summary receiver-operating characteristics (sROC) curve was constructed. Predetermined subgroup analysis was also performed.

**Results:**

Thirty-three studies were included in the final analysis, evaluating 2949 patients. The pooled sensitivity and specificity were 0.69 (95% CI 0.68–0.69) and 0.84 (95% CI 0.83–0.85), respectively, and the sROC AUC was 0.84 (95% CI 0.81–0.87). Subgroup analysis showed significantly better sensitivity with high *b*-values (> 1000 s/mm^2^). There was high statistical heterogeneity between studies.

**Conclusion:**

The diagnostic accuracy of combined DWI and T_2_WI is good with high *b*-values (> 1000 s/mm^2^) seeming to improve overall sensitivity while maintaining specificity. However, further large-scale studies specifically looking at *b*-value choice are required before a categorical recommendation can be made.

**Electronic supplementary material:**

The online version of this article (10.1007/s00261-017-1400-4) contains supplementary material, which is available to authorized users.

With a crude incidence of 134.3 per 100,000, prostate cancer is the most common cancer in men, and the second-biggest cause of cancer mortality [1, 2]. The quoted incidence has increased in recent years; however, this may be due to the use of prostate-specific antigen (PSA) blood testing. The majority of suspected cases with either a high PSA, abnormal digital rectal examination (DRE), or suggestive symptoms, will undergo a transrectal ultrasound guided biopsy (TRUS) to confirm and grade a histopathologic diagnosis [3]. If this is positive and the patient is a candidate for radical treatment, they will receive multiparametric magnetic resonance imaging (mpMRI) to assess the extent of cancer growth. However, there are now a substantial number of centers choosing pre-biopsy mpMRI followed by a more-targeted biopsy.

Multiparametric MRI is a well-established imaging modality for assessing prostate cancer, predominately to exclude extra-glandular spread and to judge how much of the prostate is involved. It consists of multiple sequences, including T_1_- and T_2_-weighted imaging (T_2_WI), diffusion-weighted imaging (DWI) and, in some instances, Dynamic contrast-enhanced (DCE) imaging. Multiple meta-analyses have proven DWI to have good diagnostic accuracy [4–6]; its contrast is governed by numerous technical parameters, one of the most important of these is the diffusion-weighting factor, or ‘*b*-value’. The *b*-value reflects the strength and timings of magnetic field gradients applied to the patient, and acquisition of multiple *b*-values permits calculation of an apparent diffusion coefficient (ADC) map, which gives a quantitative measure of tissue diffusion that has been shown to have an inverse correlation with tumor Gleason score [7]. Currently the recommendation is to use at least two *b*-values, one of 50–100 s/mm^2^, 800–1000 s/mm^2^ and if possible 1400–2000 s/mm^2^ [8, 9]. Theoretically, increasing the maximum *b*-value results in a better contrast-to-noise ratio (CNR) because there is greater suppression of normal prostate tissue signal, so resulting tumors are more apparent. However, the tradeoff is a reduced signal-to-noise ratio (SNR). Even though *b*-values > 1400 is recommended, there is little evidence supporting this and there is no widely accepted optimal “high *b*-value.” In a previous meta-analysis, Wu et al. showed no benefit from increasing *b*-value but only one paper in the analysis used *b*-values of over 1000 [4]. A multitude of recent studies have shown high sensitivity and specificity with higher *b*-values using both visual and ADC value assessments [10–12]. For clinical relevance, we hope to investigate the diagnostic accuracy achievable by visual assessment of DWI in combination with T_2_WI at high *b*-values > 1000 s/mm^2^.

## Materials and methods

This review was registered with the PROSPERO International prospective register of systematic reviews (reference number: 42016036196) prior to commencement [13]. The review was carried out in accordance with the preferred reporting items for systematic reviews and meta-analysis (PRISMA) guidance [14].

A systematic review of the literature was independently undertaken by two reviewers, who identified studies that investigated the diagnostic accuracy of DWI and T_2_WI MRI in the detection of prostate cancer. Searches were performed using MEDLINE and EMBASE electronic databases, as well as OpenSIGLE to explore sources of unpublished gray literature. The Science Citation Index was used to identify articles which cite those identified with the original search terms. Once eligible studies were found, their reference lists were manually searched for further potential papers. The search strategy for MEDLINE, including Boolean operators and MeSH terms, is presented in Table 1; the same search strategy was used for each database with alterations to suit. All studies were included up to the date of the search: 1st of September 2017.

**Table 1:** MEDLINE search terms and strategy

1	Exp prostate* neoplasm*/
2	Prostat* cancer*.mp.
3	Prostat* carcinoma*.mp.
4	Or/1–3
5	Exp diffusion magnetic resonance/
6	DW magnetic resonance imaging.mp.
7	DWI.mp.
8	DW-MRI.mp.
9	Or/5–6
10	4 and 9
11	10 Limit to human studies
12	11 Limit to english language

### Eligibility

The eligibility criteria for the studies included within the systematic review were that they used both DWI and T_2_WI MRI in combination for the assessment of prostate cancer; they were applied for the assessment of the pretreatment patient population with a histopathologic reference standard, be that biopsy or radical prostatectomy; they reported sufficient information to produce a 2 × 2 table (true positives, false positives, false negatives, and true negatives) for calculation of sensitivity and specificity; they were published in English; and they assessed more than ten individual patients. To be included, both T_2_WI and DWI sequences needed to be assessed visually, with both sequences used to assess for tumor presence rather than just for localization. The choice of scoring system, such as Likert or PI-RADS, and whether a sector-based or whole gland assessment was conducted did not affect eligibility. Articles were excluded if they did not satisfy the inclusion criteria above, or if they used a combination of imaging sequences other than DWI and T_2_WI so that individual data for the desired combination could not be extracted. They were also excluded if an ADC cutoff value was used to discriminate malignant from benign tissue as opposed to visual assessment by certified radiologists. Studies were not excluded by country of origin, age of patients or study design.

### Study identification

Initially papers were reviewed by relevancy of title and then abstract. Residual articles had their full text reviewed against the inclusion and exclusion criteria. This was also done independently by the same two reviewers. Any disagreement was solved by consensus or a third expert reviewer if necessary.

### Data extraction

The following data were extracted from each eligible study: year of publication, country of origin, patient group, number of patients, average age, and PSA, study design (retrospective or prospective) and the histopathologic reference standard used. Further information on the imaging specifications was also gathered: field strength, coil used, field-of-view, *b*-value set, and whether they visually assessed DWI source images, ADC maps, or both, for each patient. True positives, false positives, false negatives, and true negatives were also extracted for pooling results. In the case of multireader studies, the most experienced was chosen for data extraction. When insufficient data were available, reviewers manually calculated them from other reported statistics, when possible. All data extraction was independently verified by two reviewers.

### Quality assessment

The quality of the individual included paper’s methodology was assessed with the Quality Assessment of Diagnostic Accuracy Studies (QUADAS-2) tool, a validated tool specifically designed to critically appraise diagnostic accuracy studies [15]. This was also undertaken independently by two reviewers and disagreement resolved with consensual discussion consulting a third expert reviewer if a consensus could not be met.

### Statistical analysis

The sensitivity and specificity with 95% confidence intervals (CIs) were calculated for each included study using the extracted details of the 2 × 2 tables, and forest plots produced.

Initially, heterogeneity of studies was examined visually using the data extraction tables. Then, statistical analysis was performed using the inconsistency value (*I* ^2^) and Q statistics of the Chi squared value, for which an *I* ^2^ value > 50% or *p* value < 0.10, respectively, represents significant statistical heterogeneity. In these cases, a random-effects model was applied to data pooling. Pooled results for sensitivity, specificity, and diagnostic odds ratio (DOR) with 95% CIs, and a summary receiver-operating characteristic (sROC) curve were also presented.

To explore predictable sources of heterogeneity between the included studies, sensitivity and 1-specificity were plotted on an ROC plane to visually assess the presence or absence of a ‘shoulder arm’ shape, which indicates a threshold effect. This was also tested statistically with the Spearman correlation coefficient of the logit of sensitivity and logit of (1-specificity), with a *p*-value < 0.05 suggesting a threshold effect. Subgroup analysis was performed for; *b*-values (< 1000, 1000 and > 1000 s/mm^2^), field strength (1.5T and 3T), coil type (endorectal and body), method of assessment (DWI source images, ADC or both), reference standard (biopsy and radical prostatectomy), tumor zone (peripheral or transitional zone) and study design (retrospective and prospective). If possible raw data was separated from individual papers for each subgroup. Pooled sensitivities, specificities, positive and negative likelihood ratios, and meta-regression of diagnostic odds ratios were performed for these subgroups with a *p*-value < 0.05 deemed as statistically significant.

Publication bias was not assessed as there is currently no recognized or appropriate method that does so with sufficient power for diagnostic accuracy studies, and the impact of publication bias is presently unknown for studies of this type [16].

All statistical analysis was performed using Meta-DiSc (version 1.4, Javier Zamora).

## Results

### Search results

**Fig. 1:**
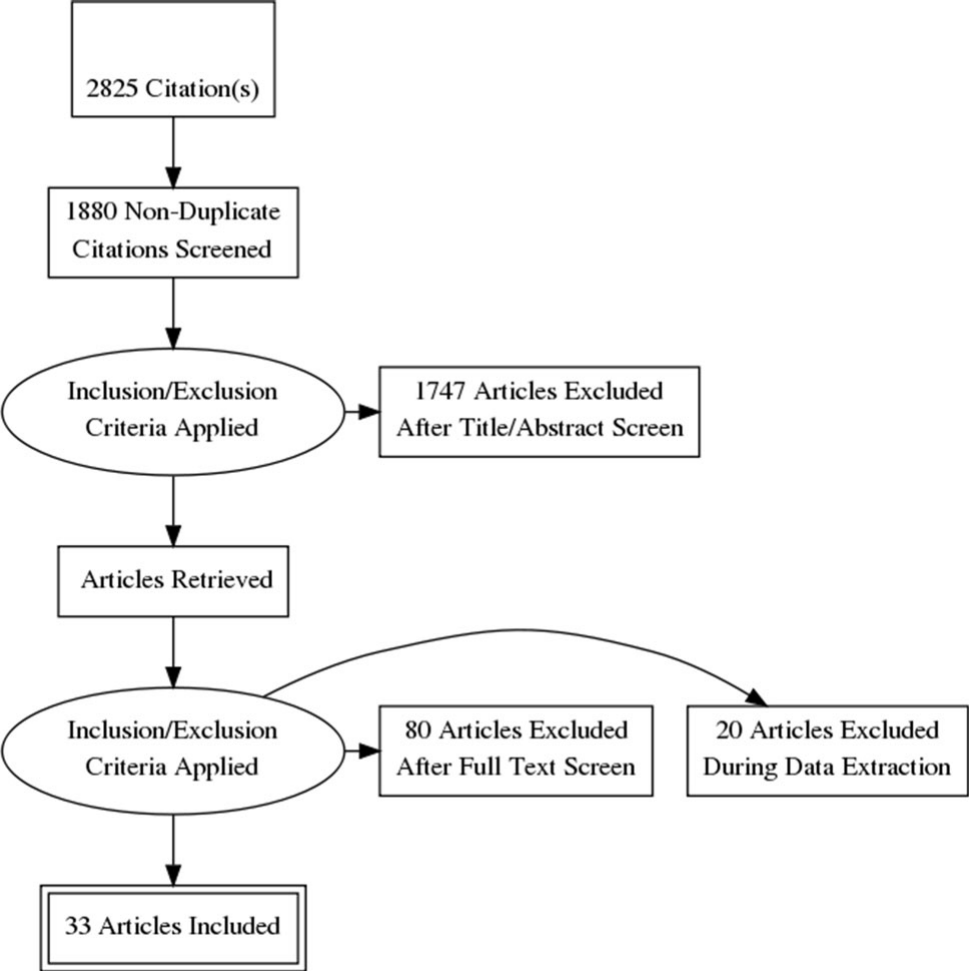
PRISMA flow diagram. ADC, apparent diffusion coefficient; DWI, diffusion-weighted imaging; T_2_WI, T_2_-weighted imaging

With the above-presented search strategy, 2825 citations were discovered, and after duplicates were removed, there were left 1880 unique articles. A total of 33 studies were included in the final analysis after reviewing against the eligibility criteria. The PRISMA flowchart of the search results is presented in Fig. 1.

### Quality assessment

The full results of the QUADAS-2 appraisal are presented in Table 2. The strengths across the included studies were that the vast majority used consecutive patient selection with appropriate inclusion and exclusion criteria. However, two studies [17, 18] limited their investigation to transitional zone tumors and another [19] to patients with ‘low risk’ cancer. Therefore a subgroup analysis was deemed particularly important to assess the differences between peripheral and transitional zone tumors. Another strength was that all index tests used were applicable to clinical practice, without any nonstandard imaging methods. All but one study imaged patients after a positive biopsy, while patients studied by Tanimoto et al. had a pre-biopsy MRI [20]. A number of studies did not state the timings between biopsy and MRI [21–24], which could have implications if the timing was too long causing a disparity between the images and histopathology correlation or too short resulting in an increased incidence of post-biopsy hemorrhage which might limit accuracy. Kitajima et al. [25] and Morgan et al. [26] reported delays between biopsy and imaging much less than the recommended six weeks [27]. The predominant weakness of included studies was applicability of the patient groups, as studies were often limited to patients who underwent radical prostatectomy. These patients tend to be younger, with a narrower range of tumor staging. However, this is acceptable to obtain a reference test with low bias.

**Table 2:** QUADAS-2 quality assessment of included studies

Study	Risk of bias				Applicability		
	Patient selection	Index test	Reference test	Flow and timing	Patient selection	Index test	Reference test
Agha [43]	✓	✓	✗	✓	?	✓	✓
Bains [44]	✓	✓	✓	✓	✗	✓	✗
Baur [45]	✓	✓	✗	?	✓	✓	✓
Brendle [46]	✓	✓	✓	✗	✗	✓	✓
Costa [47]	✓	✗	✗	?	✗	✓	✗
Doo [48]	✓	✓	✓	✓	✗	✓	✓
Haider [49]	✓	✓	✓	✓	✗	✓	✓
Hoeks [17]	✗	✓	✓	✓	✗	✓	✓
Isabaert [21]	✓	✓	✓	?	✗	✓	✓
Iwazawa [50]	✓	✓	✗	✓	✓	✓	✓
Jung [18]	✓	✗	✓	✓	✗	✓	✓
Katahira [51]	✓	✓	✓	✓	✗	✓	✓
Kim [19]	✗	✓	✓	✓	✗	✓	✓
Kitajima [25]	✓	✓	✓	✗	?	✓	✓
Kuhl [33]	✓	✓	✗	?	✓	✓	✓
Lim [52]	✓	✓	✓	✓	✗	✓	✓
Loggitsi [53]	✓	✓	✓	✓	✗	✓	✓
Morgan [26]	✓	✓	✗	✗	✓	✓	✓
Ohgiya [54]	✓	✓	✗	✓	✓	✓	✓
Petrillo [22]	✓	✓	✗	?	✓	✓	✓
Rosenkrantz [55]	✓	✓	✓	✓	✗	✓	✓
Rosenkrantz [56]	✓	✓	✓	✓	✗	✓	✓
Shimofusa [57]	✓	✓	✓	✗	✗	✓	✓
Shinmoto [58]	✓	✓	✓	?	✗	✓	✓
Stanzione [59]	?	?	✗	✓	✗	✓	✓
Tanimoto [60]	✓	?	✗	✓	✓	✓	✓
Thestrup [61]	✓	✓	✗	✓	✓	✓	✓
Ueno 2013 [12]	✓	✓	✓	✓	✗	✓	✓
Ueno 2013 [23]	✓	✓	✓	?	✗	✓	✓
Ueno 2015 [62]	✓	✓	✓	?	✗	✓	✓
Vargas [63]	✓	✓	✓	✓	✗	✓	✓
Yoshimitsu [64]	✓	✓	✓	✓	✗	✓	✓
Yoshizako [65]	✓	✓	✓	✓	✗	✓	✓

✓ Low risk; ✗ high risk; ? unclear risk

### Study characteristics

The data extracted for study characteristics are described in Tables 3, 4, and 5. There were 2949 patients across the 33 studies. The mean age (range) was 65.1 (41–86) years, and PSA was 9 (0.4–130) ng/mL, respectively. The majority of studies (*n* = 20) used a retrospective study design as opposed to prospective (*n* = 13). Most of the studies (*n* = 19) used 3T field strength, thirteen studies used 1.5 T, and one study used both. Maximum *b*-values across the studies ranged from 600 to 2000 with the majority using 1000. Nine studies used an endorectal coil. Nine studies used DWI source images for diagnosis, while seven used ADC maps and seventeen used both. Most studies (*n* = 20) used radical prostatectomy as the reference standard while seven used TRUS biopsy, two MRI guided biopsy, one transperineal biopsy and another used a mixture of TRUS biopsy and radical prostatectomies.

**Table 3:** Principle characteristics of included studies

Study	Year	Country	No. of patients	Age (range)	PSA (range)	Design
Agha [43]	2015	Egypt	20	n/a	n/a	Pro
Bains [44]	2014	Switzerland	111	64^a^ (43–82)	n/a (0.7–112.2)	Pro
Baur [45]	2016	Germany	44	66 (46–81)	12.3 (5.2–70)	Pro
Brendle [46]	2016	Germany	15	66 (52–76)	11.8 (3.3–65.4)	Pro
Costa [47]	2016	USA	49	63 (49–79)	11.2 (2.5–48.5)	Pro
Doo [48]	2012	South Korea	51	63^a^ (50–72)	11.5 (4.2–43.8)	Retro
Haider [49]	2007	Canada	49	61^a^ (46–75)	5.4^a^ (0.9–26)	Pro
Hoeks [17]	2013	Netherlands	28	n/a (45–73)	n/a (1.9–44)	Retro
Isabaert [21]	2013	Belgium	75	66^a^ (49–64)	10.4 (1.5–70.9)	Pro
Iwazawa [50]	2011	Japan	178	69 (41–86)	n/a	Retro
Jung [18]	2013	South Korea	156	59^a^ (42–75)	4.9 (0.4–93.7)	Retro
Katahira [51]	2011	Japan	201	69 (43–80)	13.2 (2.6–114)	Retro
Kim [19]	2014	South Korea	100	63^a^ (51–76)	6.5^a^ (2.2–9.5)	Retro
Kitajima [25]	2010	Japan	53	69^a^ (56–84)	11.1^a^ (4.2–112.1)	Retro
Kuhl [33]	2017	Germany	542	64.8 (42–80)	8.5 (3.2–67.5)	Pro
Lim [52]	2009	South Korea	52	65 (48–76)	10.5 (1.2–79.6)	Retro
Loggitsi [53]	2017	Greece	26	63.7 (48–73)	8.1 (2–21.9)	Pro
Morgan [26]	2007	UK	54	68 (52–80)	10 (n/a)	Pro
Ohgiya [54]	2012	Japan	73	70 (n/a)	11.7^a^ (n/a)	Retro
Petrillo [22]	2014	Italy	136	66 (n/a)	6.8 (n/a)	Pro
Rosenkrantz [55]	2011	USA	42	62 (47–76)	6.2 (1.3–32.5)	Retro
Rosenkrantz [56]	2015	USA	106	62 (56–81)	6.9 (n/a)	Retro
Shimofusa [57]	2005	Japan	37	71 (54–82)	21.8 (4.5–130)	Retro
Shinmoto [58]	2015	Japan	87	n/a (51–75)	n/a (2.8–35.2)	Retro
Stanzione [59]	2016	Italy	82	65 (n/a)	8.8 (n/a)	Pro
Tanimoto [60]	2007	Japan	83	67 (53–87)	19.4 (n/a)	Pro
Thestrup [61]	2016	Denmark	204	64.1 (45–75)	14 (2.2–120)	Retro
Ueno [12]	2013	Japan	73	67 (50–77)	9.51 (2.9–49)	Retro
Ueno [23]	2013	Japan	80	67 (50–77)	9.51 (2.9–49)	Retro
Ueno [62]	2015	Japan	31	65 (51–81)	8.6 (4.7–16.5)	Retro
Vargas [63]	2011	USA	51	56^a^ (46–74)	5.3 (0.4–62.2)	Retro
Yoshimitsu [64]	2008	Japan	37	66 (56–75)	11.9 (0.7–54.8)	Retro
Yoshizako [65]	2008	Japan	23	65^a^ (52–76)	n/a	Retro

^a^Median; N/A, not available; Pro, prospective; PSA, prostate-specific antigen (ng/mL); Retro, retrospective)

**Table 4:** Imaging and methodological characteristics of included studies

Study	Field strength	Endorectal coil	FOV (cm)	*b*-value	Reference	AS	Method
Agha [43]	3T	N	30 × 30	0, 1000	Bx	U	Both
Bains [44]	3T	N	n/a	0, 500, 1000	RP	Y	Both
Baur [45]	3T	Both	20 × 20	0, 100, 500, 1000	MR	N	Both
Brendle [46]	3T	N	27.6 × 28	50, 800	RP	U	Both
Costa [47]	3T	Both	16 × 16	0–2000	Mix	U	Both
Doo [48]	3T	N	28 × 28	0, 1000	RP	U	ADC
Haider [49]	1.5T	Y	14 × 14	0, 600	RP	U	ADC
Hoeks [17]	3T	Y	20.4 × 20.4	0, 50, 500, 800	RP	U	Both
Isabaert [21]	1.5T	N	30.9 × 38	0, 50, 100, 500, 1000	RP	U	DWI
Iwazawa [50]	1.5T	N	30 × 30	0, 1000	Bx	U	DWI
Jung [18]	1.5T/3T	Y	12 × 12/14 × 14	0, 1000	RP	U	ADC
Katahira [51]	1.5T	N	35 × 35	0, 1000, 2000	RP	U	DWI
Kim [19]	3T	N	34 × 16.8	0, 100, 1000	RP	Y	Both
Kitajima [25]	3T	N	35 × 25	0, 1000	Bx^a^	N	Both
Kuhl [33]	3T	N	21 × 21	0, 800, 1000, 1400	MR	U	Both
Lim [52]	1.5T	Y	22 × 22	0, 1000	RP	Y	ADC
Loggitsi [53]	1.5T	N	10 × 10	0, 250, 500, 750, 1000	RP	U	Both
Morgan [26]	1.5T	Y	20 × 20	0, 300, 500, 800	Bx^a^	Y	ADC
Ohgiya [54]	3T	N	35 × 35	0, 500, 1000, 2000	Bx	U	DWI
Petrillo [22]	1.5T	Y	13.6 × 16	0, 50, 100, 150, 300, 600, 800	Bx	N	Both
Rosenkrantz [55]	1.5T	N	30 × 24.4	0, 500, 1000	RP	U	DWI
Rosenkrantz [56]	3T	N	20 × 20/28 × 21.8	50, 1000, 2000	RP	U	Both
Shimofusa [57]	1.5T	N	20 × 20	0, 1000	Mix	U	DWI
Shinmoto [58]	3T	N	24 × 24	0, 1000	RP	Y	ADC
Stanzione [59]	3T	N	20 × 20	0, 400, 2000	Bx	U	Both
Tanimoto [60]	1.5T	N	36 × 36	0, 1000	Bx	U	Both
Thestrup [61]	3T	U	19 × 19	0, 100, 800, 2000	Mix	Y	Both
Ueno [12]	3T	N	45 × 45	0, 1000, 2000	RP	Y	DWI
Ueno [23]	3T	N	n/a	0, 1000, 2000	RP	Y	DWI
Ueno [62]	3T	N	45 × 36	0, 2000	RP	Y	DWI
Vargas [63]	3T	Y	14 × 14	0, 700/0, 1000	RP	U	ADC
Yoshimitsu [64]	1.5T	N	24 × 24	0, 500, 1000	RP	U	Both
Yoshizako (65)	1.5T	N	42 × 21	0, 1000	RP	Y	Both

^a^Transperineal biopsy; AS, antispasmodic; Bx, biopsy; FOV, field-of-view; Mix, mixture of Bx and RP; MR, Magnetic resonance imaging guided biopsy; N, no; RP, radical prostatectomy; T, tesla; U, unclear; Y, yes

**Table 5:** Diagnostic performance of included studies

Study	TP	FP	FN	TN	Sens	Spec	Notes
Agha [43]	10	1	5	4	0.67	0.80	
Bains [44]	73	7	7	24	0.91	0.77	
Baur [45]	14	11	0	18	0.97	0.62	Body coil
	10	7	1	21	0.91	0.75	Endorectal coil
Brendle [46]	17	2	12	149	0.59	0.99	
Costa [47]	20	19	06	73	0.44	0.79	Body coil
	76	51	22	145	0.78	0.74	Endorectal coil
Doo [48]	113	21	58	216	0.66	0.91	
Haider [49]	120	39	29	204	0.81	0.84	
Hoeks [17]	65	39	47	101	0.58	0.72	TZ
Isabaert [21]	444	79	546	731	0.45	0.90	
Iwazawa [50]	238	223	80	883	0.75	0.80	
Jung [18]	91	62	84	699	0.52	0.92	TZ
Katahira [51]	971	559	616	2669	0.61	0.83	*b*_max_ = 1000
	1162	332	425	2896	0.73	0.90	*b*_max_ = 2000
Kim [19]	17	7	22	72	0.44	0.91	
Kitajima [25]	75	19	24	306	0.76	0.94	
Kuhl [33]	138	49	9	346	0.94	0.88	
Lim [52]	199	49	28	348	0.88	0.88	
Loggitsi [53]	43	33	62	330	0.41	0.91	
Morgan [26]	64	56	78	126	0.45	0.69	
Ohgiya [54]	25	5	30	13	0.45	0.72	*b*_max_ = 500
	43	4	12	14	0.78	0.78	*b*_max_ = 1000
	42	2	13	16	0.76	0.89	*b*_max_ = 2000
Petrillo [22]	18	48	7	63	0.72	0.57	
Rosenkrantz 2011 [55]	61	29	59	103	0.51	0.78	
Rosenkrantz 2015 [56]	34	13	28	561	0.55	0.98	*b*_max_ = 1000
	46	10	16	564	0.74	0.98	*b*_max_ = 2000
Shimofusa [57]	96	11	15	56	0.86	0.84	
Shinmoto [58]	93	12	58	185	0.62	0.94	
Stanzione [59]	29	1	5	52	0.85	0.98	
Tanimoto [60]	37	6	7	33	0.84	0.85	
Thestrup [61]	65	116	3	20	0.96	0.15	
Ueno 2013 [12]	258	87	83	156	0.76	0.64	*b*_max_ = 1000
	276	79	65	164	0.81	0.68	*b*_max_ = 2000
Ueno 2013 [23]	270	119	57	194	0.83	0.62	*b*_max_ = 1000
	275	105	52	208	0.84	0.66	*b*_max_ = 2000
	272	95	55	218	0.83	0.70	*b*_max_ = c2000
Ueno 2015 [62]	101	63	20	64	0.83	0.50	*b*_max_ = 2000
	86	51	35	76	0.71	0.60	*b*_max_ = c2000
Vargas [63]	65	10	42	157	0.61	0.94	
Yoshimitsu [64]	105	29	42	46	0.71	0.61	
Yoshizako (65)	21	2	5	14	0.81	0.88	TZ

*b*, *b*-value; c, computed; FN, false negative; FP, false positive; PZ, peripheral zone; sens, sensitivity; spec, specificity; TN, true negative; TP, true positive; TZ, transitional zone

### Meta-analysis

**Fig. 2:**
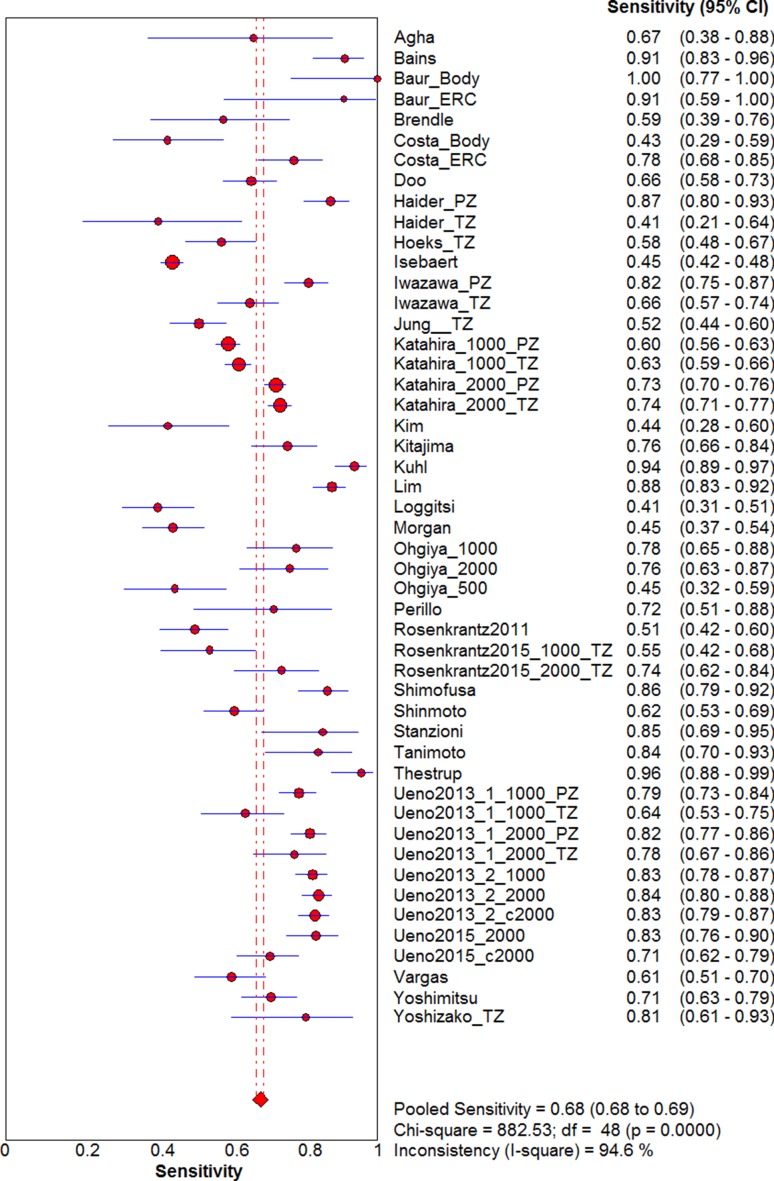
Forest plot of sensitivity for detecting prostate cancer including 95% CI, *I* ^2^ value, and *Q* statistic. CI, confidence interval; *I* ^2^, inconsistency value

**Fig. 3:**
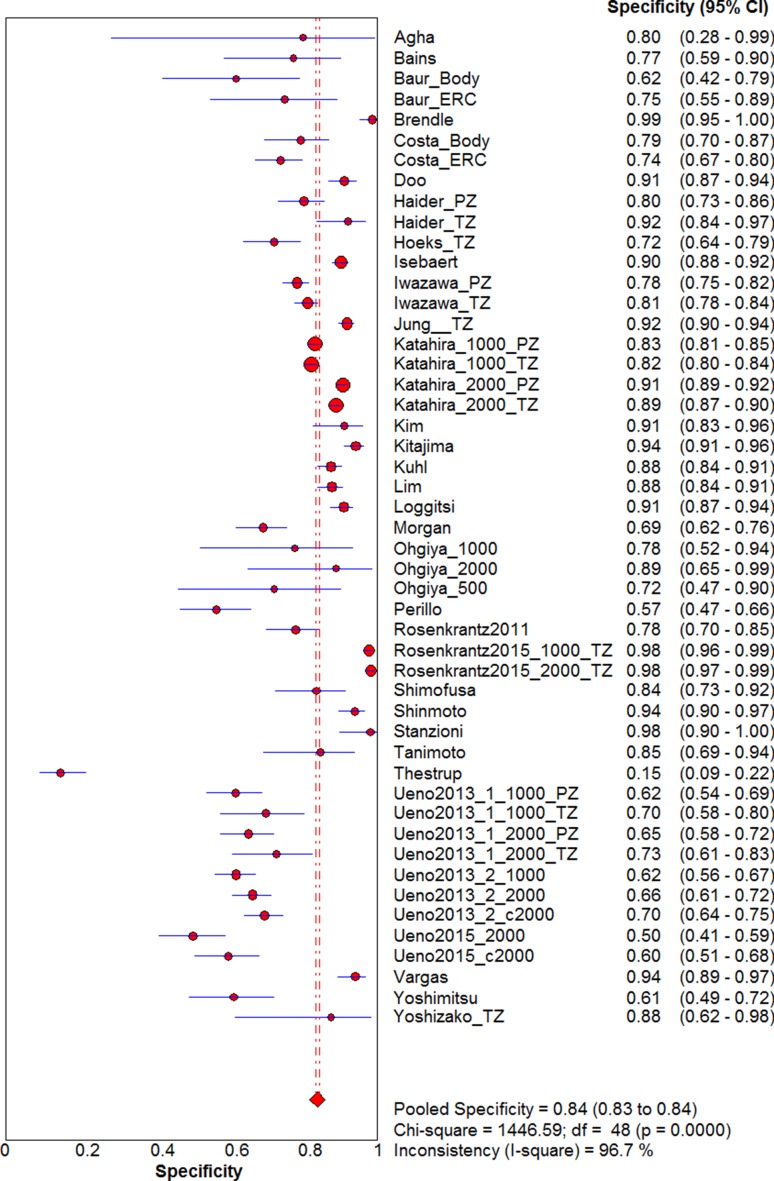
Forest plot of specificity for detecting prostate cancer including 95% CI, *I* ^2^ value, and *Q* statistic. CI, confidence interval, *I* ^2^, inconsistency value

**Fig. 4:**
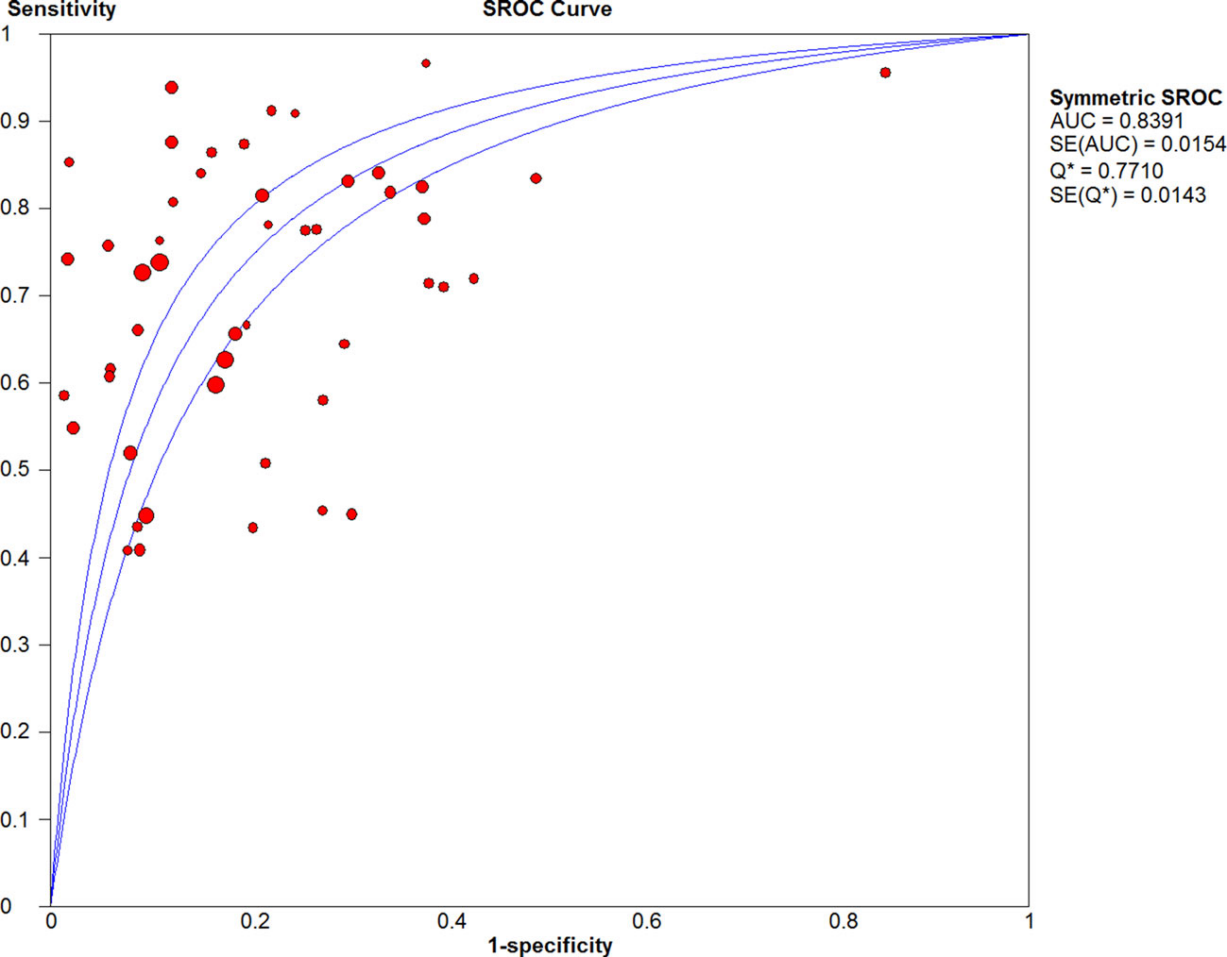
Summary receiver-operating characteristic (SROC) curve for the detection of prostate cancer. AUC, area under the curve

Visual assessment of the data extraction tables indicated they were homogeneous enough to undertake a meta-analysis with pooling. The pooled sensitivity (Fig. 2) and specificity (Fig. 3) of all included studies were 0.69 (95% CI 0.68–0.69) and 0.84 (95% CI 0.83–0.85), respectively. The pooled DOR was 12.27 (95% CI 9.60–15.68). The sROC (Fig. 4) gave an AUC of 0.839, indicating good diagnostic accuracy.

The *I* ^2^ value and Chi-square Q were 94.6% and 882.53 (*p* < 0.001), respectively, for sensitivity and 96.7% and 1446.59 (*p* < 0.001) for specificity, indicating significant statistical heterogeneity. The ROC plane (Supplementary Fig. 1) did not show a ‘shoulder-arm’ shape; however, the Spearman rank coefficient of the logit of sensitivity against logit of (1-specificity) was 0.335 (*p* = 0.018), indicating there could be heterogeneity due to a threshold effect.

### Sub-group analysis

The highest DORs were obtained when using ADC maps with or without DWI for tumors assessment and for *b*-values > 1000 s/mm^2^. Significantly higher sensitivity was achieved using *b*-values > 1000 s/mm^2^, 3T field strength, assessing PZ tumors, studies with a retrospective design and those using biopsy as a reference standard. Specificity improved significantly with a 1.5T field strength, assessing TZ tumors, using ADC maps with or without DWI and those studies using radical prostatectomy as the reference standard. The complete subgroup analysis is shown in Table 6.

**Table 6:** Subgroup analysis and meta-regression

Group (number of studies)	Sensitivity	Specificity	DOR	*I* ^2^ (%)	*P*-value
Total	0.69 (0.68–0.69)	0.84 (0.83–0.85)	12.268 (9.60–15.68)	90.7	
*b*-value					0.068
< 1000 (*n* = 7)	0.60 (0.56–0.64)	0.80 (0.78–0.83)	8.02 (3.18–20.26)	91.4	
1000 (*n* = 23)	0.64 (0.62–0.65)	0.85 (0.84–0.85)	12.56 (9.56–16.50)	86.0	
> 1000 (*n* = 13)	0.78(0.76–0.79)	0.83 (0.82–0.84)	14.32 (9.06–22.65)	92.0	
Field strength					0.418
1.5 T (*n* = 14)	0.64 (0.63–0.65)	0.85 (0.84–0.86)	10.68 (7.34–15.55)	94.0	
3 T (*n* = 28)	0.76 (0.75–0.78)	0.81 (0.79–0.82)	13.77 (9.54–19.88)	87.6	
Coil					0.597
Body (*n* = 33)	0.68 (0.67–0.69)	0.85 (0.84–0.85)	13.06 (10.05–16.97)	90.4	
Endorectal (*n* = 9)	0.68 (0.65–0.71)	0.84 (0.84–0.85)	10.40 (4.87–22.24)	93.1	
Tumor zone					0.239
PZ (*n* = 6)	0.71 (0.70–0.73)	0.84 (0.82–0.85)	12.64 (7.13–22.41)	94.4	
TZ (*n* = 11)	0.66 (0.64–0.68)	0.88 (0.87–0.88)	13.46 (8.08–22.44)	92.6	
Assessment method					0.070
DWI (*n* = 12)	0.68 (0.67–0.69)	0.82 (0.81–0.83)	8.91 (6.8–11.68)	90.2	
ADC map (*n* = 7)	0.66 (0.64–0.69)	0.89 (0.87–0.90)	15.44 (6.8–35.05)	93.8	
Both (*n* = 20)	0.72 (0.70–0.75)	0.86 (0.85–0.87)	18.58 (9.77–35.30)	90.0	
Design					0.918
Prospective (*n* = 15)	0.59 (0.56–0.61)	0.81 (0.80–0.83)	11.93 (6.61–21.54)	89.2	
Retrospective (*n* = 28)	0.71 (0.70–0.72)	0.84 (0.84–0.85)	12.56 (9.57–16.49)	91.3	
Reference standard					0.420
RP (*n* = 26)	0.67 (0.66–0.68)	0.85 (0.85–0.86)	12.09 (9.24–15.81)	91.4	
Biopsy (*n* = 13)	0.73 (0.70–0.76)	0.81 (0.80–0.83)	15.83 (7.27–34.44)	91.3	

CI, confidence interval; DOR, diagnostic odds ratio; *I* ^2^, inconsistency value; T, tesla; PZ peripheral zone; TZ transitional zone; DWI diffusion-weighted imaging; ADC, apparent diffusion coefficient; RP, radical prostatectomy

## Discussion

The findings from this study show the diagnostic accuracy of DWI and T_2_WI of prostate cancer is good when using visual assessment. The greatest diagnostic accuracy is achieved with *b*-values > 1000 s/mm^2^, and when assessing lesions with both DWI source images and ADC maps, although the interplay between sensitivity and specificity can be significantly altered by the choice of field strength and by whether tumors originate from the peripheral or transitional zone. The overall strength of the evidence on which this analysis was based was graded as good by the QUADAS-2 critical appraisal tool [15]. However, there was a high degree of unknown statistical heterogeneity, so care should be taken when interpreting these results, and even though this review cannot specify an optimal imaging protocol, it does highlight the likely important factors to be considered.

Our pooled results match those of meta-analyses investigating T_2_WI and DWI by Wu et al. and Tan et al.; this is likely due to the large overlap of included studies [28, 29]. Compared with Godley et al. and Jie et al. who analyzed the use of DWI alone, we observed a higher sensitivity but lower specificity [5, 30]. However, when we compare the results for just peripheral zone tumors, our pooled results are similar. This would suggest that the addition of T_2_WI improves the sensitivity for diagnosing transitional zone tumors; however, neither Godley nor Jie et al. presented a subgroup for TZ tumors or comparison [10]. This finding supports the present consensus that T_2_WI with DWI should be the predominant imaging protocol for diagnosing TZ tumors [9].

We observed a significant increase in sensitivity using a maximum *b*-value > 1000 s/mm^2^, and improved specificity with a maximum *b*-value of ≥ 1000 s/mm^2^. The improved contrast-to-noise ratio at higher *b*-values, resulting from the relative suppression of normal prostate tissue, would explain the increase in sensitivity by making tumors more visually apparent. Two of the studies [23, 24] also used computed high *b*-values. These synthetic data extrapolated from low *b*-value datasets showed relatively decreased sensitivity and increased specificity compared to the equivalent acquired *b*-values. There has been limited research comparing the diagnostic accuracy of computed DWI to standard DWI, but the method shows promise with reduced distortion and ghosting and improved tumor conspicuity [31, 32].

We also note that all studies using *b*-values > 1000 s/mm^2^ were limited to a maximum *b*-value of 2000 s/mm^2^, except the study by Kuhlet al [33]. Wang et al. and Metens et al. found *b*-values of 1500 s/mm^2^ gave a better tumor contrast and image quality than *b*-values of 1,000 or 2000 s/mm^2^and Kuhl et al. using a *b*-value of 1400 s/mm^2^, produced some of the highest sensitivities and specificities [33–35]. However, more data on the diagnostic accuracy of *b* ≈ 1,500 DWI are required. Furthermore, the maximum *b*-value, the minimum *b*-value, and the number of *b*-values have all been shown to have a strong influence on the calculated ADC values [36]. However, there is little evidence about their impact on diagnostic accuracy with visual assessment [10, 36].

All but two of the included studies in this analysis used *b* = 0 s/mm^2^ as the minimum *b*-value, but the number of *b*-values ranged from two to seven. Thörmer et al. found that using just two *b*-values and a minimum *b*-value of 50 s/mm^2^ gave an improved qualitative image score versus data with a minimum *b*-value of 0 s/mm^2^ [37]. However, they tested only a limited number of combinations, and used a maximum *b*-value of just 800 s/mm^2^. The significant heterogeneity of *b*-value choice in the included studies makes it extremely difficult to provide a conclusion that high *b*-values are indeed superior for diagnostic accuracy. The individual studies that tested multiple *b*-value sets on the same cohort do, however, show improved diagnostic accuracy using *b* = 2,000 as opposed to 1000 or lower. Further studies directly comparing *b*-value sets of different maximum, minimum, and a number of intermediary *b*-values would be required to make a stronger recommendation of *b*-value choice.

DOR was not significantly different between 1.5T and 3T studies (*p* = 0.418), but 3T studies showed a significantly higher sensitivity and significantly lower specificity than those performed at 1.5T. Higher field strengths have the advantage of increased SNR, which can be traded for better spatial and temporal resolutions; they also lead to increased susceptibility artifact and signal heterogeneity, and there is conflicting evidence with respect to the categorical advantage of 3T over 1.5 T [38]. There is a trend toward better diagnostic accuracy with 3T in our study, although this may be because these systems allow the use of higher *b*-values, which improve diagnostic accuracy. This result reflects the recommendations of PIRADS v2 that 1.5T and 3T are both adequate, but 3T is regarded optimal if available [9].

For a few of the studies, it was possible to separate the results for PZ and TZ, and we found significantly higher sensitivity for the PZ, but higher specificity for TZ. Often TZ tumors are of a lower grade than those found in the PZ, so they may be less apparent on imaging [39, 40]. There is also difficulty in differentiating malignancy from benign nodules common in the TZ, which are often heterogeneous and can demonstrate restricted diffusion. Along with the relative rarity of TZ tumors this may explain the drop in sensitivity but the overall DOR was not significantly different. It may be that different imaging parameters are needed for optimal diagnosis of peripheral or transitional zone disease.

Our results showed a significant increase in both sensitivity and specificity when using ADC maps with or without DWI source images for diagnostic assessment, as opposed to using DWI source images alone. There are many advantages to using ADC maps which might explain this change. Firstly, ADC maps give a quantitative measure of tissue diffusion, and are particularly useful in differentiating areas which have high signal on DWI images due to T_2_ shine-through, such as post-biopsy hemorrhage; this leads to reduced false positives and improved specificity versus weighted images. The ADC value can also be used to help confirm malignant lesions, which have low ADCs due to restricted diffusion, and this would explain the higher sensitivity seen.

Retrospective studies investigated men with previously confirmed prostate cancer, and therefore the readers knew there was cancer present in each prostate examined. This may cause the readers to be more liberal with diagnosing suspicious lesions in borderline cases where there were no other lesions in the gland, explaining the significantly higher sensitivity.

Using radical prostatectomy as the reference standard allows the assessment of individual tumors within the gland and is a more accurate method of defining tumor. TRUS biopsy is ‘blind’ and only samples a small area of the prostate, with a 20–30% false negative rate. This would lead to increased false positives on imaging, decreasing the specificity as we observe in the subgroup analysis.

This systematic review has a few limitations. Our search was, first, limited by a finite number of databases although those chosen contain the majority of the relevant journals, and by exploring the gray literature and hand-searching references, we believe the search strategy was of sufficient sensitivity. Specific databases for the research question were sought, but none existed. Second, the search was limited to the English language. The majority of articles are published in English, but there may be data in other languages that we did not include in this meta-analysis. We did not assess for publication bias for reasons stated in the statistical analysis section. The degree to which publication bias impacts diagnostic tests is unknown [16]. We did not review the exact T_2_WI parameters for the included studies, which could explain some of the heterogeneity seen. Reader experience is another factor which we did not assess as it was often poorly reported and in different formats such as years practicing, years reporting prostate mpMRI, or number of prostate mpMRIs. It is recognized that reader experience is important in interpreting mpMRI and should be considered when implementing prostate imaging [41, 42]. Although diagnostic accuracy is very important for prostate cancer assessment, there are other aims of mpMRI which have not been assessed in this meta-analysis: for example, assessment of extracapsular extension, seminal vesicle or lymph node involvement, and the ability of mpMRI to quantify tumor size and volume. These findings are all used in staging of disease and are relevant to decisions about optimal imaging sequences.

In conclusion, the diagnostic accuracy of combined diffusion- and T_2_-weighted magnetic resonance imaging for prostate cancer detection is good, and our results support the PI-RADS v2 guidelines [9]. The use of *b*-values > 1000 s/mm^2^ seem to improve the sensitivity while maintaining specificity. However, due to large amounts of heterogeneity, we cannot categorically recommend using maximum *b*-values up to 2000 s/mm^2^ for all DWI protocols for prostate cancer assessment. Further large-scale study investigating optimal *b*-value maximum, minimum, and number of *b*-values for the visual assessment of prostate cancer is required.

## Electronic supplementary material

Supplementary Figure 1. Receiver operating characteristics (ROC) plane, indicating the absence of a ‘shoulder arm’ shape. Below is the link to the electronic supplementary material.

Supplementary material 1 (JPEG 286 kb)
