# Supplementary figures and images for: The diagnostic accuracy of high b-value diffusion- and T2-weighted imaging for the detection of prostate cancer: a meta-analysis
Source: Abdom Radiol (NY). 2017 Nov 24;43(7):1787–97. doi: 10.1007/s00261-017-1400-4 (PMC6061488; doi:10.1007/s00261-017-1400-4)

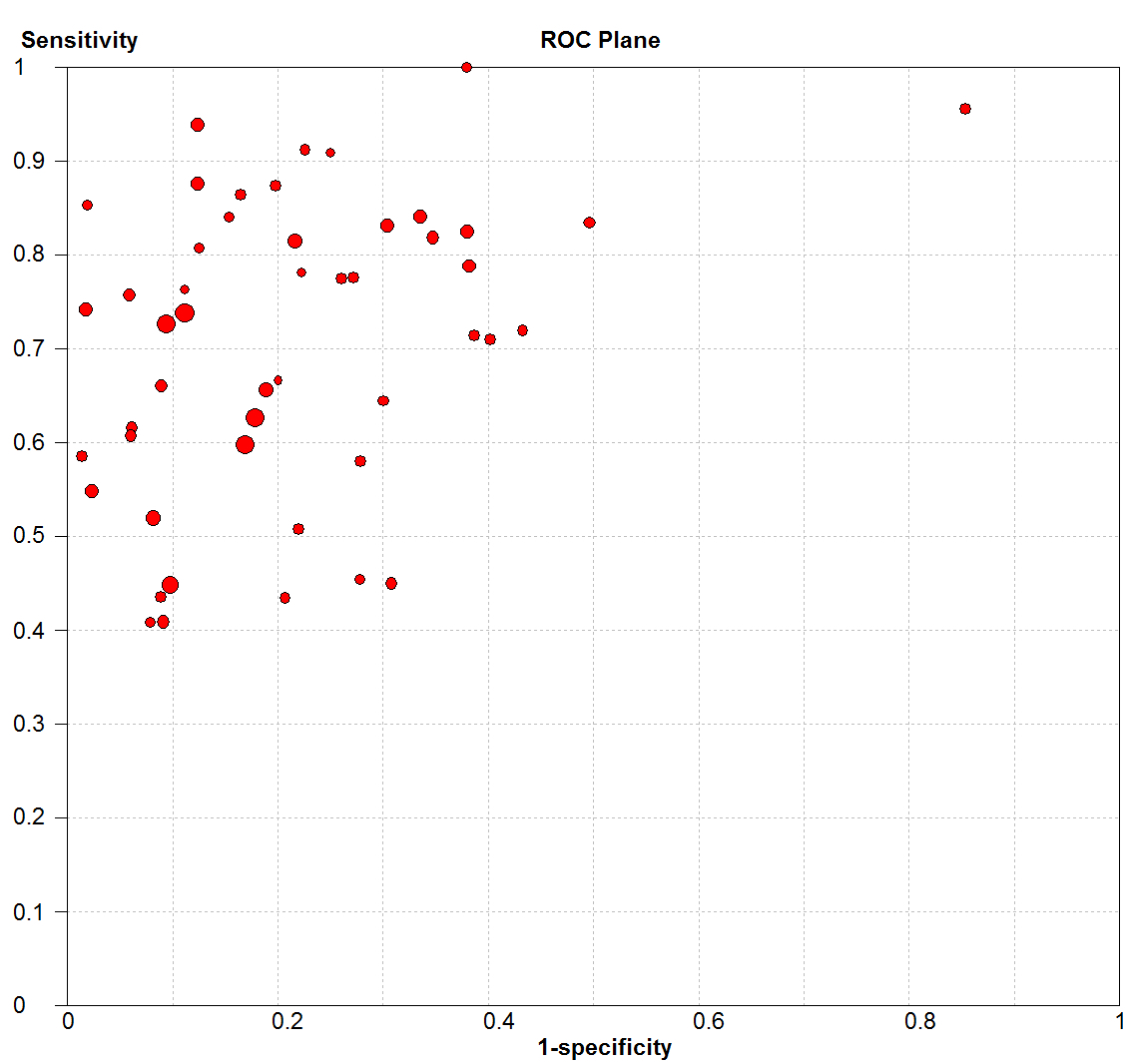

Supplement: Supplementary file 1 — Supplementary material 1 (JPEG 286 kb) [file 261_2017_1400_MOESM1_ESM.jpg]
